# Current status and prospects of nanopore sequencing technology in the detection of pathogenic microorganisms

**DOI:** 10.3389/fmicb.2026.1843102

**Published:** 2026-06-12

**Authors:** Guo Run Zi, Da-jiang Zhang, Dong-Lin He, Fan Shu, Yitian Ou, Chang-xing Ke

**Affiliations:** Department of Urology, The Second Affiliated Hospital of Kunming Medical University, Kunming, China

**Keywords:** drug-resistant gene, metagenomics, nanopore sequencing, pathogenic microorganism detection, point-of-care diagnosis, real-time sequencing

## Abstract

Rapid and accurate detection of pathogenic microorganisms is the key to clinical diagnosis and treatment as well as public health prevention and control. As a representative of the third-generation sequencing technologies, nanopore sequencing technology has brought revolutionary potential to the field of pathogen detection by virtue of its unique advantages such as long read length, real-time sequencing and portable instruments. This paper aims to review the current application status of this technology and prospect its future development. Firstly, the basic principles and the development of mainstream platforms of nanopore sequencing are outlined. Subsequently, its specific applications in the detection of various pathogens including bacteria, viruses, fungi and parasites are systematically elaborated, with a focus on analyzing the practice and remarkable advantages of this technology in scenarios such as direct metagenomic detection without culture, rapid identification of drug resistance and virulence factors, and point-of-care rapid diagnosis. Meanwhile, this paper also objectively discusses the main technical challenges faced in the current application, including the raw read accuracy, the complexity of bioinformatics analysis and the balance between cost and benefit. Finally, the future technological optimization, standardization of data analysis workflows and the expansion of broader clinical application scenarios are prospected. Importantly, this review aims to equip clinical laboratory professionals with a balanced, evidence-based framework to evaluate the readiness, utility, and implementation pathway of nanopore sequencing for specific diagnostic use-cases (e.g., urgent meningitis/endophthalmitis, culture-negative infections, resistance gene detection) within the constraints of a clinical lab, such as cost, turnaround time, and staff expertise, in order to provide new technical perspectives and theoretical support for the precise diagnosis and active surveillance of infectious diseases.

## 1 Introduction

Rapid and accurate identification of pathogenic microorganisms is the core of the diagnosis, treatment, prevention and control of infectious diseases. Traditional culture methods are time-consuming and have stringent requirements for culture conditions, making it difficult to deal with pathogens that grow slowly, are hard to culture or non-culturable (Fu et al., 2022; Deng et al., 2024). Although polymerase chain reaction (PCR)-based molecular diagnostic technologies have improved the detection speed, they usually target specific gene fragments of known pathogens, which makes it difficult to cope with unknown, rare or emerging pathogens, and also unable to fully reveal the structure of mixed infections or complex microbial communities (Deng et al., 2024; Zhang L. L. et al., 2022). In recent years, high-throughput sequencing technologies, especially nanopore sequencing technology, have brought revolutionary changes to the detection of pathogenic microorganisms. Compared to other third-generation sequencing technologies like PacBio SMRT, which offers highly accurate long reads (HiFi) but requires higher capital investment and longer turnaround times, nanopore sequencing provides superior portability and real-time data streaming, making it more adaptable for point-of-care and rapid clinical diagnostics (MacKenzie and Argyropoulos, 2023; Chen et al., 2023; Oehler et al., 2025; Petersen et al., 2019). Recent benchmarks demonstrate that the latest ONT R10.4.1 chemistry has closed the accuracy gap, achieving >99% raw read accuracy while maintaining a significant advantage in Turnaround Time (TAT; Ni et al., 2023). For time-critical scenarios like sepsis or UTI diagnostics, the “sample-to-result” integration of ONT remains the most applicable scenario compared to the centralized, high-throughput nature of PacBio platforms (Bellankimath et al., 2024; Tai et al., 2025). Based on the changes of electrical signals, nanopore sequencing performs real-time and long-read sequencing of single-stranded DNA or RNA molecules. Its equipment (such as MinION from Oxford Nanopore Technologies) has the advantages of portability and low cost, enabling its direct application in clinical laboratories and even on-site environments (Zhang L. L. et al., 2022; Chaves et al., 2025; Torres Montaguth et al., 2026).

The core advantages of nanopore sequencing technology lie in its long read length, real-time analysis and portability of equipment (Long et al., 2025). The long read length feature allows it to span repetitive regions and structural variations in the genome, enabling more accurate genome assembly and species identification (Zhang L. L. et al., 2022). Real-time analysis means that sequencing data can be processed during generation, which significantly shortens the time from sample collection to result acquisition, and this is crucial for critical infection cases requiring rapid diagnosis (Li et al., 2025; Jing et al., 2025). For example, in clinical scenarios such as endophthalmitis and intra-amniotic infection, nanopore sequencing can complete the entire process from DNA extraction to pathogen identification within a few hours (e.g., 5–12 h), which is much faster than traditional culture methods (Jing et al., 2025; Chaemsaithong et al., 2023). The portability of its equipment (such as MinION) has even broken the limitations of laboratory space, making it possible to perform sequencing in resource-limited areas, epidemic outbreak sites and even at the bedside, thus providing a powerful tool for point-of-care rapid diagnosis and public health emergency response (Kumburu et al., 2023; Bastug et al., 2025). Furthermore, in critical care settings, particularly for patients with sepsis, rapid pathogen detection is a matter of life and death. Nanopore sequencing excels in this core clinical scenario by enabling rapid identification of blood-borne pathogens and antimicrobial resistance profiles directly from positive blood cultures or plasma, significantly accelerating the initiation of targeted, life-saving antimicrobial therapies compared to culture-dependent workflows (Tai et al., 2025; Elbehiry et al., 2025; Liao et al., 2025; Ali et al., 2024; Han et al., 2024).

In clinical practice, nanopore sequencing has demonstrated excellent diagnostic performance. A number of studies have confirmed that compared with traditional microbial culture methods, nanopore targeted sequencing (NTS) or metagenomic next-generation sequencing (mNGS) has higher detection sensitivity and a wider coverage of pathogen spectrum (Fu et al., 2022; Li et al., 2025). For example, in the detection of bronchoalveolar lavage fluid from patients with pulmonary infection, the sensitivity of NTS is significantly higher than that of traditional methods, especially for the detection of specific important pathogens (such as *Mycobacterium tuberculosis*, non-tuberculous mycobacteria, fungi, etc.; Li et al., 2025; Ma et al., 2024). For culture-negative cases, nanopore sequencing can often reveal potential pathogens, including mixed infections, providing key diagnostic clues for clinical practice (Chan et al., 2020; Hao et al., 2023). In addition, this technology is not sensitive to antibiotic exposure; even if patients are receiving antibiotic treatment, its detection efficiency is not affected, while traditional culture methods are significantly hindered (Li et al., 2025). This makes it of great value in guiding the timely adjustment of anti-infective treatment regimens in clinical practice. After adjusting antibiotics based on NTS results, the condition of most patients has been significantly improved (Li et al., 2025).

Nanopore sequencing has a wide range of applications, covering various sample types from common infection sites (such as the respiratory tract and urinary tract) to sterile sites (such as cerebrospinal fluid and abscess fluid; Zhao et al., 2024). This technology has shown its ability to comprehensively and rapidly identify the spectrum of pathogenic microorganisms, including bacteria, viruses and fungi, in community-acquired pneumonia, bloodstream infection, central nervous system infection, osteoarticular infection and opportunistic infections in immunocompromised patients (such as HIV-infected individuals; Zhang et al., 2024, 2025; Nan et al., 2022). It can not only perform species identification, but also detect virulence factors and antimicrobial drug-resistant genes related to pathogens at the same time, providing a molecular basis for precise anti-infective treatment and infection control (Zhang L. L. et al., 2022; Zhao et al., 2024). For example, this technology has been successfully used to monitor the spread of carbapenemase genes (such as *blaNDM*) and reveal the multi-species outbreaks caused by plasmid horizontal transfer in hospitals (Raabe et al., 2003). Table 1 presents key clinical validation studies of nanopore sequencing for infectious disease syndromes in recent years (Sung et al., 2025; Zhang L. et al., 2022; Lee et al., 2024; Deng et al., 2022). Despite the broad prospects of nanopore sequencing technology, its wide clinical application still faces several challenges, including the relatively high error rate of raw sequencing data, the high requirement for the removal of host background nucleic acids in complex samples, the standardization of data analysis workflows and the further optimization of the cost-benefit ratio (Zhang L. L. et al., 2022; MacKenzie and Argyropoulos, 2023). However, with the continuous improvement of sequencing chemistry, nanopore sensors and bioinformatics algorithms, its accuracy and throughput are constantly increasing (MacKenzie and Argyropoulos, 2023; Wang et al., 2021). In the future, nanopore sequencing is expected to combine with microfluidics, artificial intelligence and other technologies to further realize an automated and integrated rapid pathogen detection platform, pushing the diagnosis and treatment of infectious diseases into a more precise and rapid new stage (Cui et al., 2025).

**Table 1:** Key clinical validation studies of nanopore sequencing for infectious disease syndromes.

Type of disease	Sample type	Nanopore method	Comparative method	Key findings	References
Bacterial meningitis	Cerebro-spinal fluid (CSF)	16S amplicon/mNGS	Culture +nanopore/culture	Increased detection rate (70.6% vs. 47.1%) in culture-negative cases	Sung et al., 2025
Urinary tract infection	Urine	Metagenomic nanopore sequencing (mNPS)/mNGS	Nanopore/culture	6-h TAT; accurate AMR prediction	Zhang L. L. et al., 2022
Infectious uveitis	Intraocular fluid	mNGS	Nanopore/culture	17h TAT vs. 4 days traditional methods	Lee et al., 2024
Pulmonary infection	Bronchoalveolar lavagefluid (BALF)/blood	Amplicon/mNGS	Standard care	Sensitivity 83.9% vs. 44.6%; 17.5h TAT	Deng et al., 2022

This review will comprehensively sort out and in-depth analyze the application of nanopore sequencing technology in the detection of pathogenic microorganisms from the technical basis to specific practice, and then to challenges and prospects. Specifically, this review aims to equip clinical laboratory professionals with a balanced, evidence-based framework to evaluate the readiness, utility, and implementation pathway of nanopore sequencing for specific diagnostic use-cases (e.g., urgent meningitis/endophthalmitis, culture-negative infections, resistance gene detection) within the constraints of a clinical lab (cost, turnaround time, staff expertise).

## 2 Principles and platform overview of nanopore sequencing technology

### 2.1 Core technical principles and workflow

**Figure 1:**
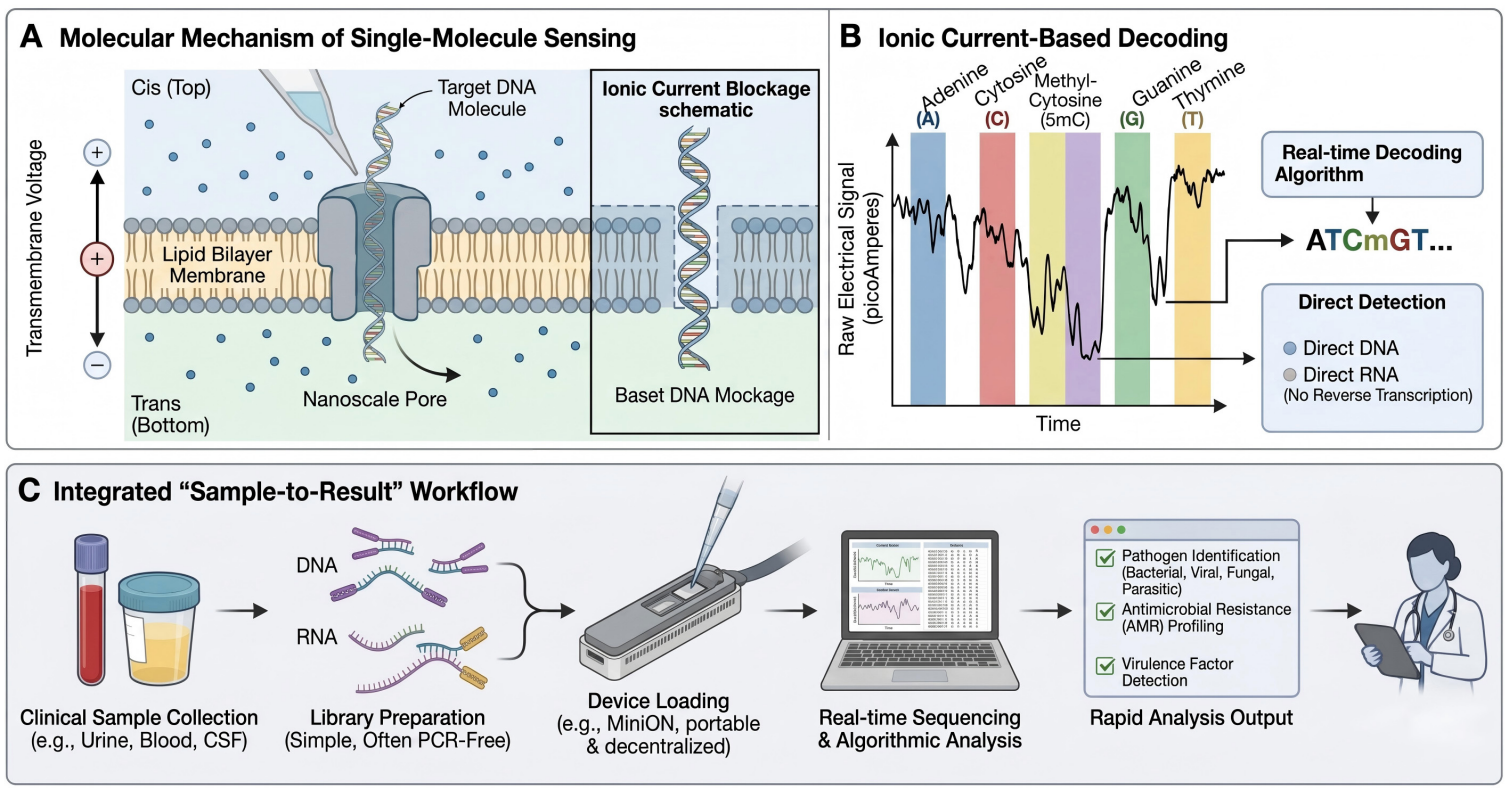
Nanopore sequencing biophysical mechanism and integrated workflow. **(A)** Molecular mechanism of single-molecule sensing: the core sensing system comprises an insulating lipid bilayer and an engineered protein nanopore (e.g., MspA, α-HL, or CsgG derivatives). Driven by a constant transmembrane voltage, the target native nucleic acid molecule in the cis chamber (cathode) is translocated through the nanopore toward the trans chamber (anode). The steric hindrance of the polymer within the pore restricts the steady-state flow of ions, generating transient ionic current blockades with base-specific amplitudes and biophysical characteristics; **(B)** lonic current-based decoding: the dynamic recording of raw electrical signals (in picoAmperes) over time is illustrated. Canonical nucleotides (A, C, G, T) and epigenetic modifications (e.g., 5-methylcytosine, 5mC) exhibit discrete current blockade levels and distinct dwell times as they pass through the sensing region of the pore. Utilizing real-time, deep learning-based basecalling algorithms (e.g., RNN or Transformer architectures), the system enables high-accuracy direct decoding of native DNA or RNA sequences, thereby eliminating the need for reverse transcription or polymerase chain reaction (PCR) amplification; **(C)** Integrated “sample-to-result” workflow: this panel illustrates the practical implementation of the technology for point-of-care testing and clinical diagnostics. The highly integrated workflow encompasses the standardized acquisition of clinical body fluid specimens (e.g., whole blood, urine, cerebrospinal fluid), optimized rapid library preparation chemistries (typically amplification-free transposase-based or rapid ligation methods), and device loading onto portable, decentralized sequencing platforms. Coupled with standardized bioinformatics pipelines, real-time data acquisition facilitates the rapid generation of comprehensive reports—including metagenomic pathogen identification, antimicrobial resistance (AMR) profiling, and virulence factor detection—providing immediate and precise data support for clinical decision-making.

The core principle of nanopore sequencing technology is based on single-molecule sensing. When a single nucleic acid molecule (DNA or RNA) passes through a nanoscale pore embedded in a thin film driven by a transmembrane voltage, it will temporarily block the ionic current and generate a characteristic current blockage signal (Ying et al., 2022). This process does not rely on DNA polymerase or fluorescent labeling, realizing true single-molecule and real-time sequencing (Lin et al., 2021). Different bases (including modified bases) produce current changes of different amplitudes and durations (i.e., blockage signals) when passing through the pore due to the differences in their physicochemical properties (such as size, shape and charge; Wei et al., 2024). The raw current signals are decoded in real time through advanced algorithms, which can be converted into the corresponding base sequences (Wang et al., 2021). From sample to clinical report, the workflow of this technology is highly integrated and rapid (see Figure 1). Library preparation is usually relatively simple; for DNA sequencing, PCR amplification is usually not required, thus avoiding amplification bias and retaining base modification information (Chen et al., 2023). After sample loading, the sequencing process is carried out in real time, and data can be analyzed while being generated, which greatly shortens the time from sample collection to result acquisition (Zhou et al., 2022). The entire process from library construction to data analysis reflects its convenience and rapidity, making it particularly suitable for application scenarios requiring rapid response, such as clinical diagnosis and epidemic monitoring (Torres Montaguth et al., 2026; Wang et al., 2021; Zheng et al., 2023).

### 2.2 Mainstream sequencing platforms and their characteristics

At present, the leading manufacturer of nanopore sequencing technology is Oxford Nanopore Technologies (ONT; Lin et al., 2021). The company provides a diversified product line to meet the needs of different throughput and application scenarios. Its portable devices MinION and Flongle are compact in size, realizing the portability and decentralization of sequencing equipment, and are particularly suitable for point-of-care rapid detection, bedside diagnosis and applications in resource-limited areas (Chen et al., 2023; Zheng et al., 2023). For research requiring higher throughput, ONT provides platforms such as PromethION, which can be combined with MinION and other technologies to optimize the effect of plant genome assembly (Cao et al., 2020). In addition to DNA sequencing, a significant advantage of this technology is its support for direct RNA sequencing without reverse transcription into cDNA, thus enabling the direct acquisition of the sequence and modification information of RNA molecules (Wang et al., 2021). While the more common cDNA-PCR approach in clinical virology is often preferred for low-titer clinical samples due to its higher sensitivity, though it requires longer preparation times, direct RNA sequencing preserves epigenetic modifications and avoids reverse transcription bias (Hewel et al., 2025). At the same time, nanopore sequencing can also directly detect epigenetic modifications [such as DNA methylation (5mC, 6mA, etc.)] during the sequencing process, providing an additional dimension for studying the epigenome of pathogens and its functions in infection and drug resistance (White and Hesselberth, 2022; Schababerle et al., 2025; Tan et al., 2024). These characteristics make the ONT platform not only perform well in genome sequencing and assembly, but also show great potential in the fields of transcriptomics, epigenomics, and rapid pathogen identification (Wang et al., 2021; Zheng et al., 2023).

## 3 Application of nanopore sequencing in the detection and typing of bacterial pathogens

### 3.1 Bacterial species identification and genome assembly

The long read length characteristic of nanopore sequencing shows significant advantages in bacterial species identification and genome assembly. Its long read length can span repetitive regions in the bacterial genome, thus realizing nearly complete *de novo* genome assembly, which is crucial for accurate identification to the species and even subspecies level (Chen and Xu, 2023). For example, in the direct detection of clinical samples, nanopore sequencing has been proven to effectively identify pathogenic bacteria that are difficult to detect or culture-negative by traditional culture methods. A study on cerebrospinal fluid (CSF) from patients with suspected bacterial meningitis showed that in culture-negative cases, 16S rRNA amplicon sequencing based on the nanopore platform could detect potential pathogenic bacteria, thus increasing the overall positive rate from 47.1% of culture to 70.6% (Sung et al., 2025). Similarly, in resource-limited environments, the combination of nanopore sequencing and conventional culture methods increased the pathogen detection rate of cerebrospinal fluid samples from 40% to 57% (Pallerla et al., 2022). This ability is particularly prominent in mixed infections or complex samples. For example, in tear samples from patients with keratitis, nanopore sequencing not only accurately identified all culture-positive pathogenic bacteria, but also detected bacterial pathogens in some culture-negative ulcers, and even successfully identified bacterial species in cases where scraping culture could not be performed due to too small ulcers, revealing pathogens that may be missed by traditional methods (Dibbs et al., 2025). Studies have confirmed that the rapid sequencing workflow based on MinION can significantly shorten the diagnosis time. In the diagnosis of urinary tract infection (UTI), the optimized metagenomic nanopore sequencing (mNPS) workflow can complete pathogen detection and antibiotic resistance prediction within 6 h after sample receipt (Zhang L. et al., 2022). For infectious uveitis, the average turnaround time from sample receipt to report of nanopore metagenomic sequencing (about 17 h) is much shorter than that of traditional methods (about 4 days; Lee et al., 2024). In immunocompromised cancer patients, the average turnaround time of nanopore amplicon sequencing is about 17.5 h, which is also shorter than that of traditional culture methods, and it shows higher sensitivity (83.9% vs. 44.6%), especially in both blood and non-blood samples (Deng et al., 2022). These studies have jointly confirmed that nanopore sequencing can realize a rapid whole process from sample to species identification, providing the possibility for timely clinical intervention.

### 3.2 Drug-resistant gene analysis and molecular epidemiological investigation

Nanopore sequencing has unique value in drug-resistant gene analysis and molecular epidemiological investigation. Its long read length can obtain the complete plasmid or genomic island sequence containing drug-resistant genes in real time, thus clarifying the genetic context of drug-resistant genes (such as mobile genetic elements upstream and downstream), which helps to track the transmission mechanism of drug resistance (Chen and Xu, 2023). For example, a study used nanopore sequencing to analyze eight multidrug-resistant (MDR) bacterial strains from clinical, environmental and food sources, successfully assembled their genomes and 18 plasmids, and revealed the composition of structurally complex plasmids and evidence of horizontal transfer of plasmids between different bacterial species through long read length analysis (Ye et al., 2024). This is crucial for understanding the transmission of pathogens in hospital-acquired infections. Through nanopore sequencing of positive blood cultures, pathogens can be identified within 10 min after the start of sequencing, and all predetermined drug-resistant genes and plasmids, including the correct identification of plasmids and *blaCTX-M* subtypes, can be detected using raw data within 1 h (Taxt et al., 2020). This ability to quickly obtain genomic information makes it possible to perform rapid whole-genome sequencing of nosocomial outbreak pathogens such as carbapenem-resistant Enterobacterales (CRE) and methicillin-resistant *Staphylococcus aureus* (MRSA), thus realizing accurate traceability of outbreak sources and reconstruction of transmission chains. For example, NanoCore is a user-friendly genome monitoring method based on nanopore sequencing data, which can calculate and visualize sample distances similar to core genome multilocus sequence typing (cgMLST) directly from raw reads. Verification results show that when only nanopore data are used, the pairwise distances between MRSA and vancomycin-resistant *Enterococcus faecalis* (VRE) strains calculated by NanoCore are highly consistent with the Illumina-based commercial gold standard method, and can produce the same clustering results, effectively supporting the monitoring of bacterial pathogens and outbreak detection in medical institutions (Fuchs et al., 2024). In addition, long read length makes multilocus sequence typing (MLST) and core genome multilocus sequence typing (cgMLST) analysis more accurate and efficient. Although the latest nanopore sequencing methods (such as R10.4.1 combined with the Dorado SUP model) have improved the accuracy of genome assembly and annotation, their resolution still needs to be specifically evaluated according to species for reliable cgMLST analysis (Purushothaman et al., 2026). A study on ESKAPE pathogens showed that in the detection of antimicrobial resistance (AMR) genes, Illumina, Oxford Nanopore Technologies (ONT) and hybrid assembly have their own advantages and disadvantages, among which hybrid assembly performs the best in identifying key AMR genetic determinants (Frias-De-Diego et al., 2025). These technologies provide powerful tools for real-time monitoring of drug resistance transmission, guiding infection control measures and optimizing the use of antimicrobial drugs.

For sepsis patients in the ICU, timely pathogen identification and targeted antibiotic intervention are key to reducing mortality rates. However, due to testing turnaround times of 24–72 h and high false-negative rates, traditional blood culture struggles to meet the demands of critical care. In recent years, nanopore sequencing technology has demonstrated transformative clinical value in this field, thanks to its real-time analysis and long read-length capabilities. The latest prospective clinical study demonstrates that by performing metagenomic sequencing directly on plasma-derived cell-free DNA, it is possible to achieve a truly “culture-free” rapid diagnosis (Nielsen et al., 2025). Furthermore, with the deep integration of automated nucleic acid extraction workstations, the process from blood sample collection to the issuance of a comprehensive report detailing pathogenic bacteria and antimicrobial resistance (AMR) genes has been successfully reduced to less than 8 working hours (Tai et al., 2025). This highly integrated rapid turnaround capability has significantly improved the success rate of early, precise treatment for patients with severe infections.

The “Accuracy vs. Speed” Trade-off in Outbreak Settings:

In the context of outbreak management and hospital infection control, laboratory directors face a strategic clinical decision regarding the “accuracy vs. speed” trade-off. For immediate infection control, prioritizing rapid, actionable data over perfectly polished genomes is often necessary (Oehler et al., 2025). A MinION sequencing run can provide highly actionable insights within just 1 h of data generation. Within this brief window, bioinformatics pipelines can deliver preliminary species identification, detect major antimicrobial resistance (AMR) genes (such as blaKPC or blaCTX-M), and effectively estimate strain relatedness using rapid core-genome surveillance tools like NanoCore (Fuchs et al., 2024). This rapid workflow allows infection control teams to immediately implement isolation protocols and adjust empirical therapies during the critical early stages of an outbreak. While the raw data from a 1-h run might lack the base-level precision of a completed dataset, it is clinically sufficient for these urgent strategic decisions. Subsequently, the diagnostic workflow can transition from a “rapid” to a “definitive” approach; as the sequencing run continues or by integrating short-read data for hybrid assembly, the laboratory can later refine the genomic picture to achieve high-resolution multilocus sequence typing and fully resolve complex AMR genetic determinants (Fuchs et al., 2024; Frias-De-Diego et al., 2025). Thus, the deployment of nanopore sequencing should be viewed as a flexible, tiered diagnostic strategy tailored to the urgency of the clinical scenario.

## 4 Practice of nanopore sequencing in viral detection and monitoring

### 4.1 Viral genome sequencing and variant tracking

By virtue of its advantages of long read length and real-time analysis, nanopore sequencing technology shows great potential in the field of viral genome sequencing and variant tracking. This technology has been widely used in the genomic monitoring of a variety of RNA viruses, such as SARS-CoV-2, influenza virus, canine distemper virus (CDV) and foot-and-mouth disease virus (FMDV; Torres Montaguth et al., 2026; Zheng et al., 2023; Lanszki et al., 2024; Abualghusein et al., 2025). Studies have shown that its direct RNA sequencing ability avoids the bias that may be introduced by reverse transcription and amplification, and can more truly reflect the original state of the virus in the sample. Although it may miss viruses with extremely low titer, the combination of rRNA depletion + cDNA-PCR protocol has achieved the same effect as Illumina MiSeq sequencing technology (Pecman et al., 2022). During the COVID-19 pandemic, nanopore sequencing based on portable devices such as MinION was widely used for real-time monitoring of the viral genome, rapid identification of key variants of concern (VOC) such as Alpha, Delta and Omicron, and accurate analysis of key mutations on the spike protein (S protein), providing key information for public health response (Pater et al., 2021; Munis et al., 2021; Zhou et al., 2022). The long read length characteristic enables nanopore sequencing to accurately resolve complex structural variations, recombination events and quasispecies distribution in the viral genome, providing a key tool for in-depth understanding of the evolutionary dynamics of viruses (Yu et al., 2025). For example, a study used a high-fidelity nanopore sequencing strategy (CLAE) to successfully resolve the quasispecies of SARS-CoV-2 in community wastewater, revealing the microevolution of the virus during transmission (Yu et al., 2025). In addition, this technology has also been used for the detection of genomic variations of live Newcastle disease virus (NDV) vaccines, realizing 24-fold genome coverage within 10 min and rapidly identifying 19 variant sites, providing an effective means for vaccine quality control (Sun et al., 2022). For DNA viruses such as monkeypox virus (hMPXV1), nanopore sequencing also performs excellently, which can quickly complete whole-genome sequencing for tracking virus introduction, transmission and the emergence of new lineages, and play an important role in global epidemic monitoring (Bosmeny et al., 2023).

### 4.2 Discovery of unknown viruses and metavirome research

In the unbiased application of metagenomic sequencing, nanopore sequencing technology has significantly improved the ability to discover new viruses from complex samples due to its long read length characteristic. Traditional short-read sequencing produces a large number of scattered short sequences in metavirome research, which brings great challenges to the splicing and identification of new viral genomes. The long read length of nanopore sequencing helps to connect these short sequences into longer, continuous viral genome fragments, and even directly obtain nearly complete viral genomes, thus improving the efficiency and accuracy of virus discovery (Yu et al., 2025; Qin et al., 2022). For example, from marine environmental samples, researchers successfully recovered novel, full-length RNA virus genomes using the CLAE strategy (Yu et al., 2025). In the monitoring of vector organisms such as ticks, metagenomic sequencing based on nanopores has been proven to have a significantly higher detection rate for a variety of tick-borne viruses such as Crimean-Congo hemorrhagic fever virus (CCHFV) and Jingmen tick virus (JMTV) than PCR methods based on amplification, and described Quaranjavirus sequences in ticks for the first time, broadening our understanding of potential zoonotic pathogens (Ergunay et al., 2023). Its real-time performance is a revolutionary advantage, allowing preliminary bioinformatics analysis to be carried out shortly after the start of sequencing, and quickly judging whether viral sequences exist in the sample, which greatly accelerates the process of pathogen identification of emerging or sudden infectious diseases (Zhu et al., 2020; Buddle et al., 2024). In the field of clinical diagnosis, such as the etiological diagnosis of endophthalmitis or uveitis, nanopore metagenomic sequencing can quickly and accurately identify potential pathogens in culture-negative samples, including viruses, bacteria and fungi, showing its strong ability in the diagnosis of complex infections (Lee et al., 2024; Low et al., 2022). In terms of blood transfusion safety monitoring, as an unbiased method, nanopore metagenomic sequencing can comprehensively detect blood-borne pathogens in plasma samples, including known and unknown viruses, and realize strain-level identification, providing a new supplementary strategy for ensuring blood safety (Ragupathy et al., 2025). These applications indicate that nanopore sequencing is becoming an indispensable viral detection tool in environmental monitoring, vector organism research and clinical infectious disease prevention and control.

## 5 Exploration of nanopore sequencing in the detection of fungi, parasites and other pathogens

### 5.1 Fungal infection and drug resistance detection

Nanopore sequencing technology shows significant advantages in the rapid diagnosis of fungal infections and detection of drug resistance. For clinically important fungi such as *Candida* and *Aspergillus*, this technology can not only realize rapid species identification, but also detect gene mutations related to drug resistance of antifungal drugs such as azoles. A number of clinical studies have confirmed its application value. A study on lower respiratory tract fungal infections showed that metagenomic third-generation sequencing (mTGS) based on the nanopore platform could detect 11 kinds of fungi including *Pneumocystis jirovecii, Cryptococcus neoformans*, and *Aspergillus fumigatus*, while traditional culture could only identify six kinds, and missed pathogens such as *Pneumocystis jirovecii* and *Talaromyces marneffei* (Liu et al., 2025). The study proved that mTGS had a sensitivity of 78.1% and a specificity of 90.5%, and shortened the detection turnaround time from 2 to 7 days of traditional culture to 7 h significantly (Liu et al., 2025). In another study on fungal endophthalmitis, nanopore metagenomic sequencing successfully identified the pathogenic pathogens in all eight patients, while traditional methods only confirmed five cases, and its overall turnaround time from sample to result (average 17 h) was much shorter than that of traditional methods (average 4 days; Lee et al., 2024). The long read length characteristic of nanopore sequencing makes it have a unique advantage in resolving the highly repetitive rDNA gene cluster in the fungal genome, thus improving the resolution of species identification and effectively distinguishing species with close genetic relationship. For example, by targeted sequencing of the fungal ribosomal internal transcribed spacer (ITS) and D1–D3 regions, researchers can quickly (4.92 days faster than culture on average) detect rare human pathogens including *Trichosporon asahii* and *Basidiobolus ranarum*, and effectively distinguish *Candida* and non-*Candida* infections (Kim et al., 2024). In addition, a study on the *Cryptococcus* species complex showed that nanopore sequencing had a consistency of up to 99.83% with Sanger sequencing by sequencing the fungal intergenic spacer (IGS), and could complete sequencing within 1 h, providing a new strategy for rapid molecular diagnosis (Morrison et al., 2020). These studies indicate that nanopore sequencing, with its fast, high-resolution and comprehensive detection capabilities, is becoming a powerful tool for the diagnosis of fungal infections and drug resistance monitoring.

### 5.2 Parasite identification and genomic analysis

The application of nanopore sequencing technology in the field of parasitology has brought innovation to species identification, drug-resistant gene detection and genomic research. In the detection of Plasmodium, this technology can realize the integration of species identification, drug-resistant gene detection and population genetic analysis. A study established a new method based on recombinase-aided isothermal nucleic acid amplification (RAA) combined with nanopore sequencing for the identification of *Plasmodium vivax, Plasmodium ovale, Plasmodium malariae*, and *Plasmodium falciparum* (Lin et al., 2025). This method successfully amplified the 18S rRNA genes of four kinds of Plasmodium in 49 dried blood spot samples from malaria patients, and its sensitivity, specificity and accuracy of species identification (92.00%, 97.33%, and 96.00%, respectively) were all higher than those of the traditional real-time fluorescent quantitative PCR method, providing a highly sensitive and specific supplementary means for the detection of Plasmodium (Lin et al., 2025). In addition, the long read length characteristic of nanopore sequencing is crucial for the genomic research of parasites with large and complex genomes (such as *Leishmania* and *Schistosoma*), helping to improve reference genomes and carry out comparative genomic analysis. For example, a study used nanopore sequencing to characterize *Trypanosoma cruzi* infection in *Triatoma sanguisuga* collected from dog kennels in Texas, USA. Genotyping of the mini-exon gene by quantitative PCR and nanopore sequencing revealed that all positive samples were infected with TcI type, some were mixed infections of TcI and TcIV, and successfully identified a variety of blood meal sources including dogs, humans and wild animals, providing important insights for understanding the transmission dynamics of Chagas disease in this area (Hernandez et al., 2025). Another study used nanopore sequencing to report the complete mitochondrial genome of *Armillifer moniliformis* from a patient in northern Thailand for the first time, with a full length of 16,367 bp containing 37 genes. This study provides new molecular markers for parasite species diagnosis, treatment monitoring and epidemiological monitoring (Pataradool et al., 2025). These application examples show that nanopore sequencing technology, with its long read length, real-time analysis and portability, is pushing parasite identification and genomic research into a faster and more accurate new stage.

## 6 Technical challenges, optimization strategies and future prospects

### 6.1 Technical challenges

#### 6.1.1 Main technical challenges faced at present

Although nanopore sequencing technology shows great potential in the field of pathogenic microorganism detection, its move toward large-scale clinical application still faces a series of technical challenges. Firstly, the raw read accuracy is one of the core bottlenecks. Despite the continuous progress of sequencing technology, the raw accuracy of single read length (about 95–97%) is still significantly lower than that of mature second-generation sequencing platforms (Dabernig-Heinz et al., 2024). This inherent error rate, especially when dealing with homopolymer regions, poses a substantial obstacle to the detection of low-frequency mutations, accurate identification of point mutations in drug-resistant genes or resolution of highly similar pathogen genomes, which may affect the accurate judgment of infection sources and transmission chains (Huang et al., 2022). However, this core technical bottleneck has recently been overcome. The latest R10.4.1 flow cells, combined with V14 chemistry and the Dorado neural network basecalling algorithm, have stably increased raw simplex read accuracy to over 99%, with duplex accuracy exceeding 99.9% (Helsmoortel et al., 2026). Following this leap in fundamental accuracy, recent multicenter clinical studies from 2024 to 2026 have further validated the high reproducibility and clinical reliability of this technology for pathogen genotyping and targeted resistance prediction across different laboratories (Dabernig-Heinz et al., 2024; Cottingham et al., 2026). Consequently, the current challenges for its comprehensive clinical implementation have shifted from basic sequencing accuracy to the establishment of standardized operating procedures (SOPs) and clinical-grade quality control systems across multiple centers. Secondly, the complexity of data analysis and bioinformatics workflows constitutes another major challenge. This technology can generate massive data streams in real time, which requires efficient and user-friendly analysis workflows and powerful computing resources at the back end (Ameur et al., 2019). When deployed in clinical sites or remote areas with limited resources, how to quickly and accurately parse pathogen information from raw signals and convert the results into clinically actionable reports is a major obstacle to popularization and application (Tamames et al., 2024). Thirdly, clinical sample preparation and high host background interference are difficult problems in practical application. In common clinical samples such as blood and respiratory secretions, the proportion of host (human) nucleic acids is extremely high, while the nucleic acid content of target pathogens (such as bacteria and viruses) may be extremely low (Consensus Group Of Experts On Application Of Metagenomic Next Generation Sequencing In The Pathogen Diagnosis In Clinical Moderate And Severe Infections et al., 2020; Gu et al., 2019). How to efficiently enrich trace pathogen nucleic acids without significantly increasing costs and time, or develop algorithms that can acutely identify weak pathogen “signals” from strong host background “noise,” is the key to improving detection sensitivity (Slunečko et al., 2025; Quek and Ng, 2024). Finally, the lack of a standardization and quality control system for nanopore sequencing limits the repeatability and comparability of the technology. At present, from sample pretreatment, library construction, sequencing run to data analysis, there is a lack of unified laboratory operation procedures, data analysis standards and strictly clinically verified guidelines. This insufficient standardization makes it difficult to directly compare the results between different laboratories and different studies, and also hinders its approval as a standardized diagnostic tool by regulatory agencies and its wide clinical adoption (Butler et al., 2025; Expert consensus on innovation and standardized application of nanopore sequencing technology, 2025).

#### 6.1.2 Practical implementation hurdles in clinical laboratories

Moving toward large-scale clinical application, nanopore sequencing faces distinct implementation hurdles. First, the bioinformatics burden: While integrated platforms exist, there is a need to distinguish between open-source tools (e.g., EPI2ME, What's In My Pot) which offer flexibility but require significant computational expertise, versus commercial clinical analysis suites that prioritize ease of use, validation compliance, and automated reporting (Brunet et al., 2025; Esberg et al., 2024). Second, cost-benefit analysis: The consumable cost per run, the capital cost of instruments (MinION vs. GridION vs. PromethION), and the expensive nature of host depletion kits must be meticulously weighed against standard-of-care methods like culture, multiplex PCR, or NGS send-outs (Bellankimath et al., 2025). Third, standardization and quality control (QC): Establishing unified laboratory operation procedures is critical. Clinical labs must implement internal QC protocols, including the use of control strains, continuous monitoring of sequencing yield and Q-scores, and establishing strict thresholds for reportable results to ensure reproducibility (Butler et al., 2025; Expert consensus on innovation and standardized application of nanopore sequencing technology, 2025; Abdel-Glil et al., 2025; Kan et al., 2024). Finally, biological risks: Cross-contamination in low-biomass clinical samples and the significant false positive/negative risks associated with low-abundance pathogen detection amidst high host background remain core obstacles that necessitate rigorous pre-analytical optimization (Brunet et al., 2025; Shinge et al., 2025).

### 6.2 Technical optimization and solutions

To address the above challenges, researchers and manufacturers are promoting the development of technical optimization and solutions from multiple levels. In terms of improving the quality of raw data, continuous technological iteration is the key. The developers of nanopore sequencing technology enhance the reliability of detection and the ability to distinguish subtle genomic differences by constantly updating hardware and software (Zhang et al., 2024). To solve the problem of high host background, targeted enrichment strategies have become effective solutions. By combining nanopore sequencing with CRISPR-Cas system or liquid probe capture technology, the genome of target pathogens or key regions of drug-resistant genes and virulence factors can be specifically enriched before sequencing (Wang et al., 2025). This method can significantly reduce the proportion of host nucleic acids, improve the sequencing depth of target sequences, thus realizing sensitive detection of pathogens in low-biomass samples and analysis of drug-resistant genes with higher cost-effectiveness. To lower the threshold of data analysis, the development of an integrated analysis platform is crucial. These nanopore sequencing analysis platforms encapsulate complex steps such as sequence alignment, species identification, drug-resistant gene prediction and phylogenetic analysis into a simple visual operation interface, which greatly simplifies the data analysis process, enabling clinicians or on-site technicians without profound bioinformatics background to independently complete the transformation from data to reports, and promoting the sinking and application of the technology (Tamames et al., 2024).

### 6.3 Future application prospects and development directions

Looking forward to the future, with the gradual breakthrough of technical bottlenecks and the accumulation of clinical evidence, nanopore sequencing is expected to open up broader application prospects in the field of pathogenic microorganism detection. The primary direction is to move toward the standardization of clinical diagnosis. With the continuous improvement of sequencing accuracy and the completion of more prospective, multicenter clinical verification studies, nanopore sequencing is expected to transform from a research tool into a standardized auxiliary diagnostic tool for laboratory confirmation, especially for difficult, critical or mixed infection cases. Its ability of rapid and comprehensive pathogen identification and drug resistance analysis will directly guide precise anti-infective treatment (Nielsen et al., 2025). Secondly, taking advantage of its portable equipment and real-time sequencing, it is possible to build a real-time public health monitoring network. Taking COVID-19 as an example, deploying miniaturized sequencing equipment at ports, community clinics and even remote areas can establish distributed pathogen monitoring nodes, realize early warning of infectious disease outbreaks, real-time tracking of variants and dynamic monitoring of changes in drug resistance spectra, providing real-time data support for public health decision-making (Krishna et al., 2020). The third important direction is to develop an integrated intelligent diagnostic system. The future development trend is to deeply integrate nanopore sequencers with microfluidic chips and automated nucleic acid extraction and purification modules to build a fully automated and integrated pathogen detection system with “sample in, result out”(Raymond-Bouchard et al., 2022). This system can minimize manual operation, improve detection throughput and stability, making it more suitable for clinical scenarios such as emergency departments and ICUs. Finally, the potential of this technology may go beyond traditional genome sequencing. Further developing its ability to directly detect RNA modifications, proteins and even small molecules can provide valuable multi-omics perspectives for understanding pathogen-host interactions, immune escape mechanisms of pathogens and metabolic changes during infection (Huang et al., 2022). For example, direct sequencing of pathogen RNA and identification of its modification status may reveal new gene regulatory mechanisms. In short, nanopore sequencing technology is in a stage of rapid development and integrated innovation. By continuously overcoming technical challenges and expanding application boundaries, it is expected to play an increasingly core role in the future diagnosis, treatment and prevention system of infectious diseases (Wang et al., 2021; Xu et al., 2024).

Recent multicenter clinical evidence from 2024 to 2026 solidifies its transition from a research tool to a standardized auxiliary diagnostic assay (Brunet et al., 2025; Yang et al., 2026). To realize this, we propose specific “Future Clinical Diagnostic Pathways”:

Pathway 1 (ICU Pneumonia): Bronchoalveolar lavage fluid (BALF) sample → automated microfluidic extraction with host depletion → 6-h MinION run → integrated AI pipeline reporting: (1) Dominant pathogen(s), (2) Key resistance markers (e.g., mecA, blaCTX-M), and (3) Epidemiological link to prior ICU isolates (Wang et al., 2021; Bellankimath et al., 2025).

Pathway 2 (Public Health Outbreaks): Point-of-care sequencing in a regional laboratory during an outbreak → real-time data upload to a cloud-based, standardized analysis hub → immediate public health notification of strain type and resistance pattern (Elbehiry and Abalkhail, 2025; Russell et al., 2025).

## 7 Conclusion

As a disruptive molecular diagnostic tool, nanopore sequencing fundamentally reshapes pathogenic microorganism detection by shifting the paradigm from static workflows to dynamic, real-time “sample-data-result” streams. With fundamental accuracy bottlenecks largely resolved by recent chemical and algorithmic iterations, the technology has successfully transitioned from a proof-of-concept research tool to a clinically viable platform across diverse infectious disease scenarios. It fills critical diagnostic gaps by providing rapid, portable, and comprehensive pathogen and antimicrobial resistance profiling, especially in time-critical and resource-limited settings.

Looking forward, the true potential of nanopore sequencing extends beyond basic genomic analysis; its future impact will be driven by cross-integration with cutting-edge technologies. Combining the platform with artificial intelligence for direct phenotype prediction, microfluidics for automated “sample in, result out” bedside diagnostics, and direct epigenetic detection will forge a highly intelligent and flexible molecular sensing platform.

Rather than replacing existing high-throughput short-read technologies, nanopore sequencing serves as a powerful, synergistic complement. To fully realize its potential as a cornerstone of the global public health defense system, future efforts must prioritize data standardization, the establishment of clinical interpretation guidelines, and sustained multidisciplinary collaboration among clinicians, biologists, and engineers.
